# Assessment of Limestone Waste Addition for Fired Clay Bricks

**DOI:** 10.3390/ma15124263

**Published:** 2022-06-16

**Authors:** Gyorgy Thalmaier, Nicoleta Cobȋrzan, Anca-Andreea Balog, Horia Constantinescu, Andrei Ceclan, Mirela Voinea, Traian Florin Marinca

**Affiliations:** 1 Faculty of Materials and Environmental Engineering, Technical University of Cluj-Napoca, 28 Memorandumului Street, 400114 Cluj-Napoca, Romania; gyorgy.thalmaier@sim.utcluj.ro (G.T.); traian.marinca@stm.utcluj.ro (T.F.M.); 2 Faculty of Civil Engineering, Technical University of Cluj-Napoca, 28 Memorandumului Street, 400114 Cluj-Napoca, Romania; anca.balog@dst.utcluj.ro (A.-A.B.); horia.constantinescu@dst.utcluj.ro (H.C.); ing.mirelavoinea@gmail.com (M.V.); 3 Faculty of Electrical Engineering, Technical University of Cluj-Napoca, 28 Memorandumului Street, 400114 Cluj-Napoca, Romania; andrei.ceclan@ethm.utcluj.ro

**Keywords:** fired clay brick, limestone waste, circularity, characterization

## Abstract

Our aim was to investigate the feasibility of using limestone waste resulting from stone processing for the manufacturing of fired clay bricks. Waste materials were considered as a partial replacement for clays to reduce the exploitation of natural resources and as a response to the climate neutrality commitments. The samples were prepared to have a waste content of up to 15% and were fired at a temperature of 900 °C. The chemical and mineralogical composition and the physical analysis of raw materials were investigated by using SEM–EDS and XRD diffraction. The result showed an increase in CaO in the clay mixture due to the presence of limestone, which reduced the shrinkage of the products’ compressive strength, up to 55% for samples with a higher content of limestone (15 wt.%), and influenced the samples’ color by making them lighter than the reference sample.

## 1. Introduction

The quarry and processing of natural stone yearly generate a high quantity of waste, which can be recycled in construction materials to sustain circularity and economic growth. Waste resulting from the processing of stone as formed, cladding, floor-paving elements represents up to half of the volume of the extracted material [1]. This has a significant economic and environmental impact, as well as a huge potential in recovering the construction industry [2,3] as a circular economy measure.

Studies conducted in recent years by academic and scientific researchers have highlighted the potential of using recycled limestone waste as a binder [3,4,5,6,7] or aggregate in cementitious materials [3,8,9,10], or as a filler in bituminous concrete [11], glass fiber [12], or production of ceramic materials [13,14,15]. The limited studies performed thus far on clay bricks with the addition of limestone waste have increased the interest in studying the potential of local waste recovery in the production of ceramic materials. Substituting recycled limestone waste in the clay matrix may contribute to the preservation of natural resources and reduction in waste disposal. Waste materials’ circularity resulting from the construction industry is essential in the transition toward a circular economy. The waste hierarchy “reduce–reuse–recycle” [16,17] was considered a sustainable action plan [18,19,20] to reduce the environmental impact, provide new jobs, and limit the taxes associated with pollution, including CO_2_ emissions certificates. Recovery of waste materials for manufacturing of new products should be managed by considering the waste stream, recycling process (collection, distribution, sorting, grinding, transportation), and investment costs required for a supply chain.

The quality of units is a very important parameter. Therefore, the maximum percentages of waste that can be incorporated into clay matrix must be established in accordance with the raw materials’ characteristics and green- or fired-product properties. Limestone waste may be used as a fluxing material to reduce the clay plasticity or as a pore former, enhancing the thermal characteristics of ceramic materials by increasing their porosity. The recovery potential of other waste materials rich in calcite as an admixture in the mass of clay materials has also been investigated [21,22,23].

Limestone is a material rich in calcium carbonate and can contain magnesium carbonate [24], which has been used in the past as masonry stone units for the construction of residential, cultural, and administrative buildings. Now, its use as a unit in masonry buildings is limited to ecological constructions, mainly wall cladding or floor paving. Limestone is locally available and may be used as a primary or secondary raw material (resulting from stone processing or quarries activities) for manufacturing new materials.

During the firing process, the limestone decomposition must be complete to ensure brick quality. According to [25], carbonates with particle sizes larger than 5 mm may have negative effects if used as additives in a clay matrix. The CaO grains in the presence of water may favor the appearance of portlandite [26], followed by an increase in volume [25] and a decrease in material properties.

In that respect, this paper presents our findings from experiments on fired-clay materials having a composition of up to 15% limestone waste. The aim of the present investigation was to evaluate the feasibility of the use of limestone waste as a resource for manufacturing of fired bricks. Characterization of the samples was performed in terms of mineralogical, physical, and mechanical properties.

The paper is structured in four sections. In the second section, the materials and methods used in the experimental study for investigations of raw materials and green and fired samples, are presented. In the third section, the results obtained are highlighted, and some observations and remarks are discussed to support the findings. Conclusions and further research are drawn at the end of this paper based on the results.

## 2. Materials and Methods

The raw materials were treated (dried at a temperature of 90 °C and ground), and their density was determined by pycnometry, resulting in the following values: 2.38 g/cm^3^ clays and 2.44 g/cm^3^ limestone. Based on material densities, the specimens were prepared as admixtures of clays and limestone waste resulting from stone processing activities, in different percentages by weight. The limestone content in the clay matrix was 0% (reference sample C0), 5% (sample C1), 10% (sample C2), and 15% (sample C3). The specimens were cylindrically shaped with an 18 mm diameter. Then, they were dry-pressed at 39 MPa by using a hydraulic press and fired at a temperature of 900 °C/2 h [27,28].

Scanning electron microscopy and energy dispersive X-ray spectroscopy analysis (Jeol 5600 LV microscope) were used to investigate the limestone and fired samples. For each value presented in the paper, a minimum of 5 different measurements were conducted and averaged. The density of a sample was determined by dividing the sample’s mass by the calculated volume. Because it had a regular shape, the sample’s volume was calculated from linear measurements made with 0.1 mm accuracy. Linear shrinkage was calculated as the ratio of the sample’s diameter shrinkage divided by the initial diameter, and is presented as a percentage; the linear measurements were conducted again with 0.1 mm accuracy. Similarly, the mass loss was calculated by dividing the measured weight loss from the sample’s initial mass. The values are given as a percentage. The sample’s color was taken from the sample images. Phase identification was conducted using X-ray diffraction (Equinox D3000 diffractometer), using Co-kα radiation. Phase composition was estimated from the X-ray diffraction patterns based of the height of the most intense peak for each compound. The compressive strength of the specimens was determined on cylindrical specimens with a diameter of 18 mm and an aspect ratio of 1 by using a Controls Advantest 9 hydraulic press. The load rate we used was 0.2 MPa s^−1^, and the accuracy of the pressure recording was 0.01 MPa.

## 3. Results and Discussion

### 3.1. Raw Materials

**Figure 1:**
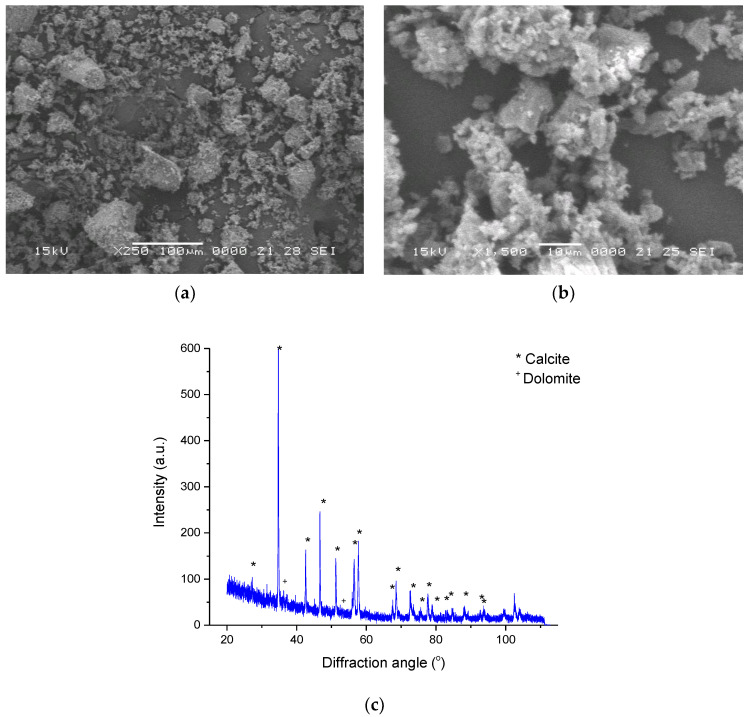
SEM images of limestone at low (**a**) and higher (**b**) magnification; X-ray diffraction pattern of the used powder (**c**).

The SEM images at different magnification and X-ray diffraction pattern are presented in Figure 1. The SEM images (Figure 1a,b) highlight an agglomerated powder with a wide size range starting from ~1 to 100 µm.

The X-ray diffraction pattern (Figure 1c) shows that limestone waste mostly contained calcite and some traces of dolomite. An X-ray diffraction peak shift was observed due to the differences in ionic radii between the main element (calcium) and the other coexisting elements.

The limestone waste was analyzed by using the EDX microprobe, and the main metals are evidenced in Table 1.

The analysis showed that limestone mainly consisted of calcium carbonate (CaCO_3_) and traces of dolomite (MgCO_3_), which may have contributed to the increase in the specimen’s porosity during firing and decrease in its density. It is noteworthy that 44 wt.% of CaCO_3_ and 52 wt.% of MgCO_3_ were emitted into the atmosphere in the form of CO_2,_ an unwanted greenhouse gas, during the firing.

### 3.2. Brick Sample

**Figure 2:**
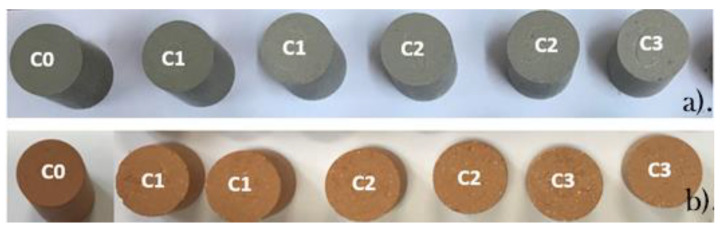
Green (**a**) and fired samples (**b**).

As in reference [25], in samples with limestone content (more highlighted in samples C2 and C3), the presence of unreacted CaO grains was observed (Figure 2b). This can generate decay of materials in the presence of water and lead to the transformation of calcium oxide (CaO) into portlandite (Ca(OH)_2_).

A higher temperature may be required for sealing CaO particles in a clay matrix to avoid this undesirable phenomenon, which increases the energy demand, the product cost, and pollution. Other issues have been highlighted [29,30], clearly showing a higher release of CO_2_ from raw materials rich in carbonate.

The lower content of Fe_2_O_3_ in samples with a higher content of limestone influenced the sample’s texture and color (Figure 2). We found that the color of a sample with the addition of limestone waste (C1–C3) was influenced by the increase in waste content, because it was lighter than the reference sample (C0).

**Figure 3:**
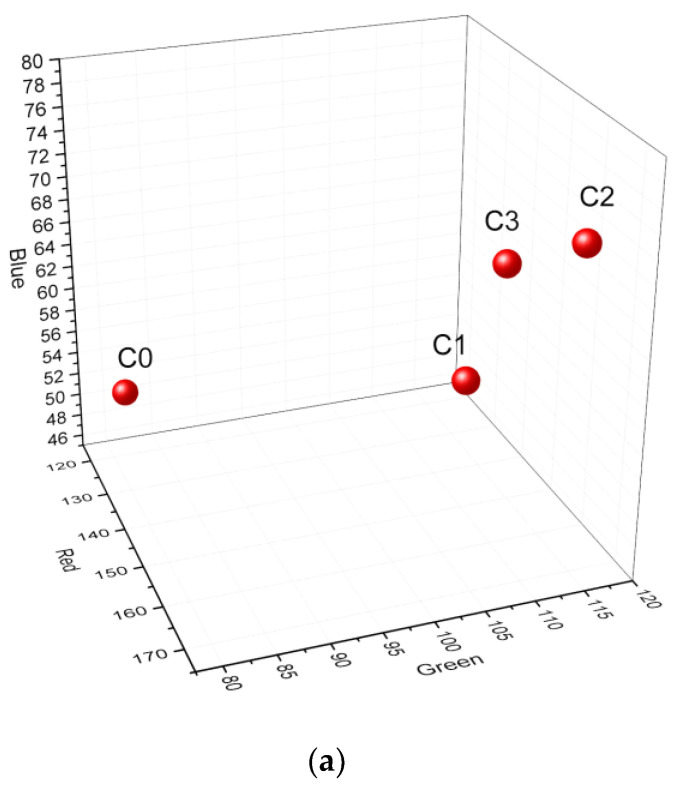

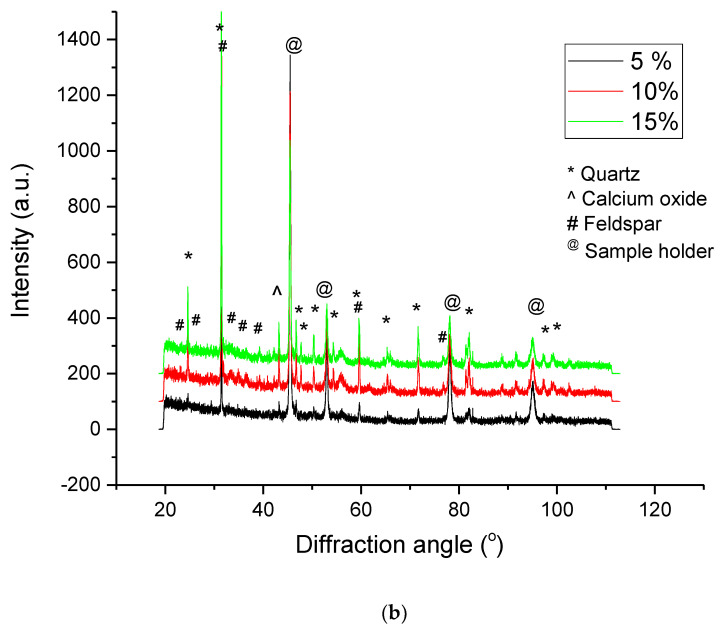
Representation of the samples color in the RGB space (**a**) and X-ray diffraction pattern (**b**).

For the description of the color changes due to the presence of limestone waste, the most employed color space (the RGB color space) was used. The RGB color space is based on the mixture of three primary, reference colors: red, green, and blue (R, G, and B). They form the base vectors of a three-dimensional orthogonal (color)–vector space, where the origin represents black because any color can be viewed as a mixture of the three. The three components are determined by the measured intensities of visible light in the long-wave (red), middle-wave (green), and short-wave (blue) area. As shown in Figure 3, which represents the samples color in the RGB space, increases in the green and blue components were recorded, so the color was lighter than that of the reference sample.

Regarding fired clay materials, the main oxides that influenced the samples color were alumina, iron oxide, calcium, and magnesium oxide. As we showed, adding the limestone into the clay matrix increased the calcium and magnesium oxide contents, while other oxides appeared to remain constant. The main components identified by X-ray diffraction were quartz (around 80%), feldspar, and calcium oxide (Table 2). Minor components (not marked in Figure 3b to avoid being illegible) were hematite, gehlenite, and muscovite.

From Table 1, one can conclude that some part of the calcium oxide remained as a free mineral present in the fired samples in the form of white spots (see Figure 2b). Some of it reacted with aluminum oxide and quartz to form at lower content gehlenite or at higher CaO content with Ca-containing minerals of the feldspar group. Based on the work of R. Kreimeyer [31], the increased CaO and MgO contents of more than 10% caused the iron oxide (responsible for the usual brown color) to be partially consumed by the formation of different compounds such as dicalcium ferrite, gehlenite, or pyroxene. From these minerals, only the gehlenite was identified in the samples with more than 10% limestone waste. This effect, the lightening of the brown color, is clearly evidenced in Figure 3, where the colors of samples C2 and C3 are far from that of the reference sample; a strong increase in the blue (short-wave) and green (middle-wave) intensities is clear. For sample C1, an increase in the green component is visible. This slight color modification compared with the reference sample was mainly due to the presence of free, white CaO particles, visible in Figure 2b. 

**Figure 4:**
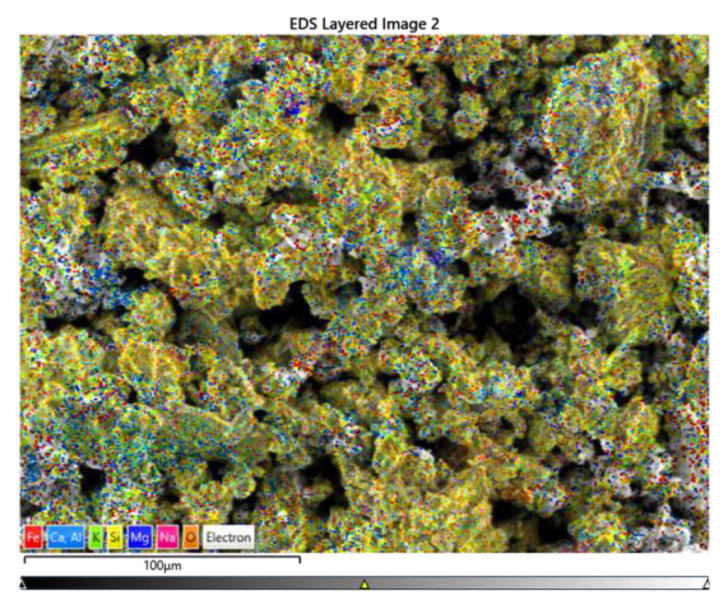
Sample EDS distribution maps (for sample C2).

Using the EDX analysis, we found three main elements whose oxide could be responsible for the color change: aluminum, calcium, and iron. The calcium was observed as individual grains in accordance with the previous observations, as presented in Figure 2 (the white dots) and further evidenced in Figure 4, and its effect was visible in the present case. The alumina and iron ratio was relatively constant for the fired samples; however the absolute value of the iron concentration steadily decreased with the increase in the limestone addition (from ~5.5 to 4.1 wt.%).

**Figure 5:**
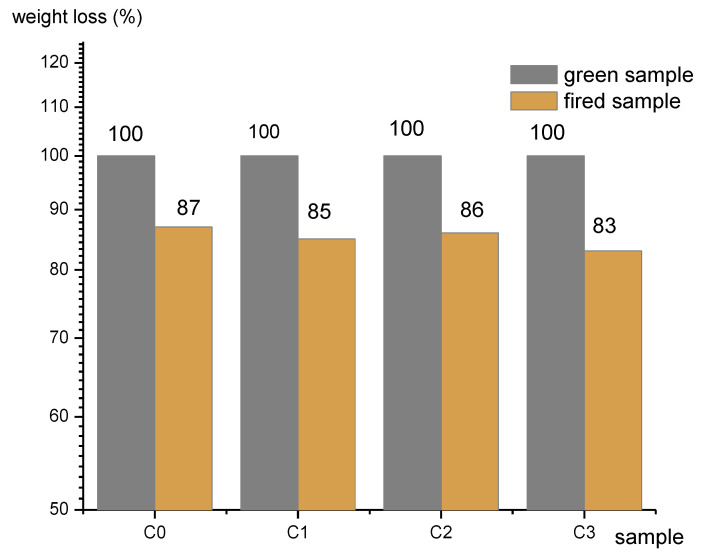
Weight of green and fired clay samples.

The masses of the green and fired samples are shown in the Figure 5. Up to 17% mass loss on firing was measured in the sample with highest content of limestone (C3), which occurred due to the thermal decomposition of inorganic substances of raw materials.

During the decomposition of carbonates, clays and limestone were transformed into CaO and CO_2_ for calcite, while the MgCO_3_ was transformed into MgO and CO_2_; both reactions require elevated temperature. The samples’ mesoporous structure [32] increased the samples’ porosities. At a lower temperature, the water was released into atmosphere due to dehydration and dehydroxylation of clay materials. CaO may react with SiO_2_, resulting in wollastonite, which may contribute to reduction in shrinkage [30].

**Figure 6:**
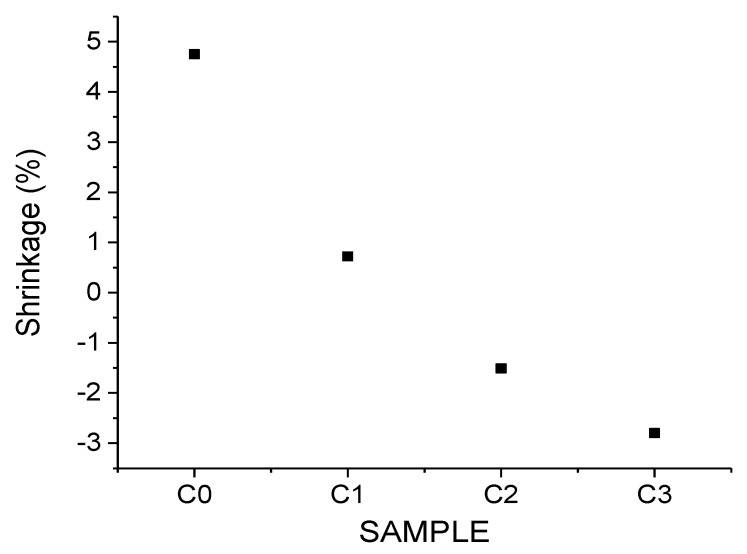
Variation of linear dimension with the limestone content.

The variation in the linear dimension with the limestone content is presented in Figure 6.

According to [33,34], shrinkage takes place due to the loss of chemically bound water during the drying and firing stage of the product because of the reduction in porosity during sintering and the expansion caused by the voids generated during the burning of organic matter and other pore formers.

The shrinkage (percent), determined as the ratio between the sample’s diameter measured before and after sintering, highlighted a decrease to 0.7% in sample C1 and a further expansion to 1.5% for sample C2 and 2.8% in sample C3. This suggested that the decrease in shrinkage depended on the increase in limestone content and was an exponential decay. Similar trends were highlighted by authors [25] in a different study.

**Figure 7:**
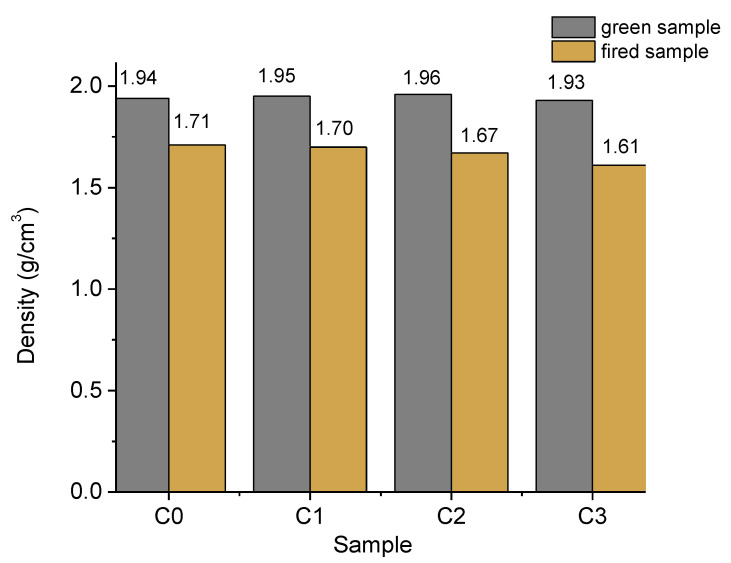
Density of green and fired samples (g/cm^3^).

The densities determined for green and fired samples are presented in Figure 7. It is shown that the green samples’ densities had insignificant increases of 1.03% (sample C2) and 0.52% (sample C1), as well as a decrease of 0.52% (sample C3) compared with the reference sample (sample C0). After firing, the density of the samples decreases from 12% in the reference sample and up to 17% in sample C3. It may also be observed that the sample density of fired bricks with the highest percent of limestone waste (C3) experienced a decrease of about 6% compared with the control sample (C0). Because the densities of the raw materials were close, their decreases are attributed solely to the increase in porosity by carbonate decomposition.

The samples with limestone as an admixture (C1–C3) were materials with high density (>1 g/cm^3^), normally being proper for masonry, and are protected against water with cladding materials if the firing temperature is lower than 1000 °C.

**Figure 8:**
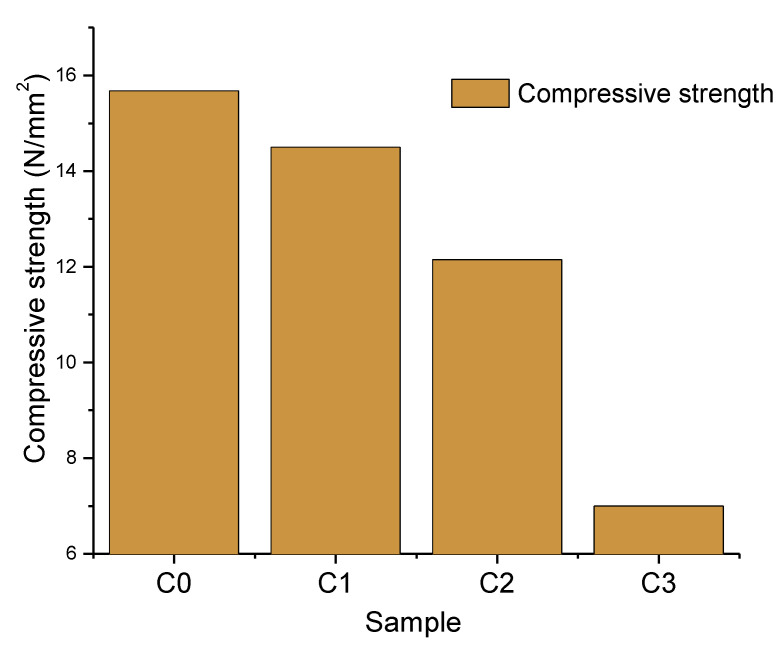
Compressive strength of samples.

The compressive strength of the fired clay samples is shown in Figure 8.

Figure 8 shows that mechanical strength of the samples was reduced by up to 55% from 15.7 to 7 N/mm^2^, such as sample C3, compared with the standard sample. Based on the results obtained, sample C3 is recommended for the production of solid units for exploitation in dry environments. Increasing the firing temperature to 1100 °C, the compressive strength of samples was reduced up to 8% in the reference samples and 9% in sample C2. By incorporating different wastes, we deviated from optimal composition, so a decrease in the mechanical properties may be expected.

To improve the mechanical characteristics of materials with limestone as an admixture, further research can be performed based on these results. Future investigation should envision the environmental impact of the materials by partially or totally considering the uptake of the CO_2_.

**Figure 9:**
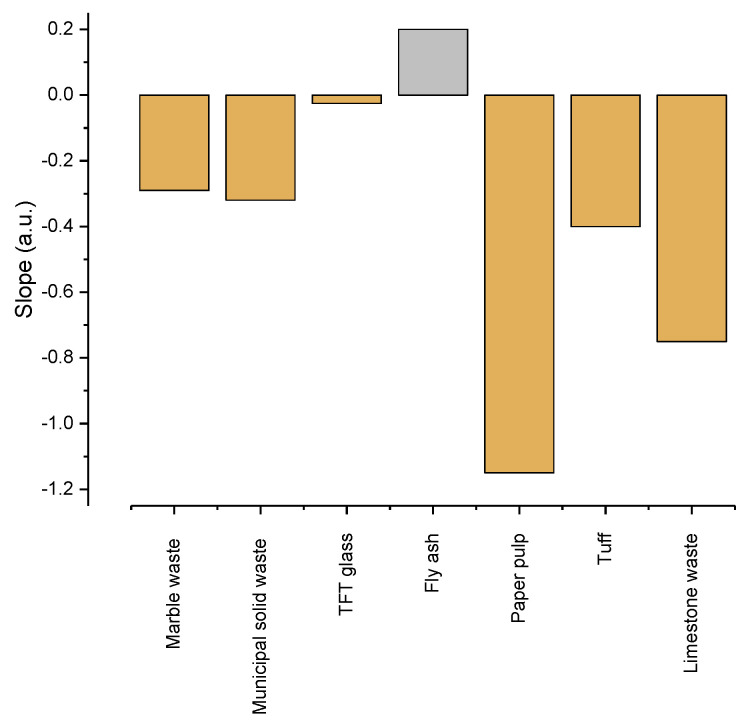
Linear decrease in compressive strength as function of used waste (marble waste [35]; municipal solid waste [36]; TFT glass [37]; fly ash [38]; gypsum waste [39]; tuff [28]; limestone waste—present work).

In order to compare the magnitude of the reduction in the compressive strength, the slope of the linear regression of the data presented in Figure 9 [28,35,36,37,38,39] was compared with the data in the literature of other commonly used waste. The positive slope indicated that the waste increased the compressive strength. As can be observed, only the fly ash had a positive effect on the compressive strength. The limestone waste had the largest negative effect. We suggest that the maximum amount of limestone waste to be used in combination with the high CO_2_ emissions is around 5%.

### 3.3. Advantages and Disadvantages of Reusing Limestone Waste as Admixture in Clay Materials

The durability of materials is one of the most important parameters to ensure the maintenance of a product in economy for many years after manufacturing, considering that waste prevention is a priority in the Waste Framework Directive [17]. Usually, clay materials have a high lifecycle compared with other building materials if they are properly used and maintained in construction. After building demolition or dismantling, the clay materials may be reused in other construction if their parameters are adequate, or they may be recycled as a binder or aggregate for new material productions.

Some opportunities for and threats of using the limestone waste as an admixture in the clay matrix are summarized in Table 3.

It is well-known that to determine the environmental impact of a manufacturing process of ceramic materials, the carbon emissions generated during exploitation of materials, technological flux (firing and drying), and decomposition of organic raw materials must be considered [40]. In the analyzed samples, the content of CaO was increased due to the addition of the limestone waste.

We propose that the contribution of limestone waste as a pore-forming agent may counterbalance the impact upon environment. This may contribute to manufacturing products with improved thermal properties and lower density.

## 4. Conclusions and Further Research Development

The results obtained on clay materials with admixture of limestone waste highlighted the following:

- The limestone waste increased the carbonate content and CO_2_ release due to their decomposition, contributed to the waste circularity, and may improve the thermal performance of products;
- The compressive strength of the samples with limestone waste was lower than that of the reference samples, with up to 55% (sample C3);
- The sample density was reduced only by 17% for the sample with highest limestone content, so it can be recommended for use in dry environments; to avoid their damage, the color of the samples lightened as the limestone waste content increased.

A holistic approach at the macrolevel is necessary in making decision regarding the stream flux and opportunity in waste recovery. Selective collection and treatment programs are needed in each country and cities with relevant action plans established for waste management based on “reuse–recycle–recovery”.

## Figures and Tables

**Table 1:** Elemental metal composition of limestone (wt.%).

Element	Mg	Al	Si	K	Ca	Fe
Content (wt.%)	2.7	3.8	7.2	1.0	82.4	2.8

**Table 2:** Estimated concentration of present minerals.

	5% Limestone Waste	10% Limestone Waste	15% Limestone Waste
Quartz	80%	82%	75%
Feldspar	8%	7%	13%
Calcium oxide	5%	6%	7%
Hematite	4%	2%	2%
Muscovite	3%	1%	1%
Gehlenite	0%	2%	2%
Total	100%	100%	100%

**Table 3:** Advantages and disadvantages of using limestone in the clay materials.

Advantages	Disadvantages
A1: limestone waste generated from stoned processing is a locally abundant resources that may partially substitute the clays in brick production or other materials A2: is a natural material that is not hazardous A3: stone processing companies may create durable partnerships with industries A4: materials industry can take advantage of financial support to sustain waste circularity and consequently limit waste disposal A5: may represent a valuable resource, especially in areas scarce in clays A6: facilitate new jobs in recycling process and revitalization of disposal areas	D1: limited knowledge about limestone recycling opportunities D2: tests on a real scale product have been insufficient performed D3: costs of primary resources are still low, which make waste processing cost-ineffective and less attractive D4: lower mechanical properties for high content in comparison with conventional materials D5: lack of chain for distribution of waste D6: may increase production cost due to waste transport and pretreatment D7: strong competition with other low-cost green materials D8: lack of guidelines for secondary raw materials
